# NT-3/TrkC Axis Contributes to the Perineural Invasion and the Poor Prognosis in Human Salivary Adenoid Cystic Carcinoma

**DOI:** 10.7150/jca.33635

**Published:** 2019-10-15

**Authors:** Huan Li, Zihui Yang, Weiqi Wang, Jun Wang, Jianying Zhang, Junye Liu, Tao Yang, Yaowu Yang, Jianhua Wei, Delin Lei, Xinjie Yang

**Affiliations:** 1 State Key Laboratory of Military Stomatology and National Clinical Research Center for Oral Diseases, and Shaanxi Clinical Research Center for Oral Diseases, Department of Oral and Maxillofacial Surgery, School of Stomatology, Fourth Military Medical University, Xi'an 710032, China; 2 Department of Radiation Medicine, Fourth Military Medical University, Xi'an 710032, China

**Keywords:** NT-3, TrkC, salivary adenoid cystic carcinoma, Schwann cells, perineural invasion

## Abstract

The present study was aimed to investigate the role and mechanism of neurotrophin-3 (NT-3) and its specific receptor tropomyosin receptor kinase C (TrkC) in the perineural invasion (PNI) process of the salivary adenoid cystic carcinoma (SACC). The co-cultured system between SACC cells and Schwann cells (SCs) was employed to detect the expression of NT-3 and TrkC. The results of ELISA, qRT-PCR and western blot showed that NT-3 was noticeably elevated in the co-cultured SACC-83 cells, while TrkC was increased in the co-cultured SCs. The results of scratch wound healing, migration, and 3D co-culture assays showed that the directional migration abilities of the co-cultured SACC-83 cells and SCs were significantly increased. Under the stimulation of NT-3, the directional motor ability of SACC-83 cells and SCs was significantly improved, and the apoptosis of SACC-83 cells and SCs were obviously inhibited. In addition, blocking TrkC by its specific inhibitor AZD7451 could significantly inhibit these effects. Immunohistochemistry staining showed that the positive expression of NT-3 (88.5%) and TrkC (92.3%) was significantly correlated with the PNI in SACC specimens (*P* < 0.05). Additionally, the high expression of NT-3 was significantly associated with the poor prognosis of SACC patients (*P* < 0.05). The present study indicated that NT-3/TrkC axis contributed to the PNI progression and the poor prognosis of SACC via regulating the interaction between SACC cells and SCs. Interruption of the interaction between SACC cells and SCs by blocking the NT-3/TrkC axis might be an effective strategy for anti-PNI therapy in SACC.

## Introduction

Salivary adenoid cystic carcinoma (SACC) is recognized as one of the major salivary gland malignancies, which accounts for about 30% of salivary gland malignant neoplasms 1. Perineural invasion (PNI) is the notorious biological characteristic of SACC that distinguishes it from other head and neck tumors. SACC patients with PNI usually present severe symptoms, including pain, numbness, facial paralysis, and lingual paralysis 2. The PNI of SACC not only destroys the structure and function of the affected nerves but also becomes a new mechanism responsible for tumor diffusion and metastasis 3. Although a series of research have focused on exploring the factors related to the PNI of SACC, the clear pathogenesis has not been fully elucidated. Therefore, a better understanding of the biological characteristics of PNI is the key to improving the prognosis of SACC patients.

Recent studies indicated that tumor microenvironment around the nerves promoted the progression of PNI in multiple nervophilic malignancies 4. SCs is the crucial factor involved in the creation of tumor-favorable conditions in the tumor microenvironment 5. Our previous studies revealed that the BDNF/TrkB axis promoted the progress of PNI via inducing the epithelial mesenchymal transformation and the SCs-like differentiation of SACC cells 6. It has been demonstrated that SCs have a unique and peculiar affinity for SACC cells 5. In addition, a recent study found that in contrast to the traditional assumption, the migration of SCs occurs firstly in the progress of PNI in pancreatic cancer and colon cancer 7. This unique phenomenon showed that besides the unilateral attack of tumor cells on the nerve, the early participation of SCs also played extremely crucial roles in the progress of PNI. Thus, we hypothesis the interaction between SACC cells and SCs might play critical roles in the progress of PNI in SACC.

Previous studies have demonstrated that neurotrophin-3 (NT-3) and its specific receptor tropomyosin receptor kinase C (TrkC) are overexpressed in pancreatic cancer and prostate cancer with PNI 8, 9. In addition, emerging evidence indicates that NT-3 can inhibit the myelination of SCs by activating TrkC to facilitate the migration of SCs 10. Activation of the NT-3/TrkC axis leads to the activation of the downstream Ras, Erk1/2, Akt, and Bcl2 signaling pathways, which ultimately enhance the invasion of SACC 11. However, the role and mechanism of the NT-3/TrkC axis in the progress of PNI in SACC have not been thoroughly demonstrated. The results of the present study for the first time indicated that the NT-3/TrkC axis promoted the PNI progress and the poor prognosis of SACC through regulating the interaction between SACC cells and SCs.

## Materials and Methods

### Cells culture

Salivary adenoid cystic carcinoma cell line SACC-83 was obtained from Peking University School of Stomatology (Beijing, China), and primary SCs obtained from the sciatic nerve of neonatal SD rats (1-3 days) by trypsin digestion. Primary SCs were separated and purified by the method of differential adherence. Cells were cultured in RPMI-1640 medium supplemented with l0% fetal bovine serum in the 5% CO_2_ incubator at 37^°^C.

### Co-culture of SACC cells and SCs

The transwell co-culture system (Corning Costar, 0.4 μm, USA) was used for the co-culture of SACC cells and SCs. SACC-83 cells and SCs were seeded into the lower chamber or the upper chamber, respectively. Then cells were co-cultured in serum-free RPMI-1640 for 48 h.

### Enzyme-linked immunosorbent assay (ELISA)

The content of NT-3 in the medium of each group was performed with the Human NT-3 DuoSet^®^ ELISA Kit (R&D Systems, Minneapolis, MN) according to the manufacturer's protocol.

### qRT-PCR analysis

Takara MiniBEST Universal RNA Extraction Kit (Takara Bio, Inc., Otsu, Japan) was used to extract total RNA. Epoch ultramicro microplate spectrophotometer (BioTek Instruments, Inc., Winooski, USA) was utilized to detect the concentration and purity of the samples. Reverse transcription was performed with the PrimeScript^TM^RT Master Mix (Takara Bio, Inc.). The qRT-PCR (BIBBY PrimeQ, UK) was carried out with the SYBR® Premix Ex TaqTMⅡ (Takara Bio, Inc.). The relative expression level of the genes was calculated by the ∆∆Ct method. The species-specific primers for the detection of NT-3 and TrkC and the internal reference β-actin were presented in Table 1.

### Western blot analysis

Total proteins were extracted from each sample. Antibodies of NT-3 (GeneTex, Inc., Texas, USA, GTX38542, 1: 5,000), TrkC (GeneTex, Inc., Texas, USA, GTX54858, 1: 2,000), and β-actin (GeneTex, Inc., Texas, USA, GTX109639, 1: 2,000) that could detect both human and rat proteins were used for the detection. The Chemidoc^TM^ XRS system with Image Lab^TM^ software (Bio-Rad Laboratories, Inc.) was utilized to visualize the protein bands, along with quantitative analysis by the Quantity One 4.4.0 software.

### Scratch wound healing assay

SACC-83 cells were seeded into the lower chamber of the transwell plates (0.4 μm), while the upper chambers were seeded with blank, NT-3 (PeproTech Inc., USA, 20 ng/ml), SCs, or SCs treated with TrkC inhibitor AZD7451 (AstraZeneca Pharmaceuticals, Waltham, MA, USA, 3 nM). Cells in the lower chamber were scratched and pictures were taken to measure the moving distance after 24 hours.

### Migration assay

The transwell system (Corning Costar, 8 μm, USA) was used for the migration abilities examination. SACC-83 cells (5×10^4^/cm^2^) and SCs (5×10^4^/cm^2^) were seeded respectively into the upper or lower chambers. Cells without migration were removed after 24 hours. The chamber was fixed with 95% ethanol and stained with crystal violet solution. Five independent visual fields were randomly selected under an inverted microscope, and the number of cells that passed through the transwell membrane was counted.

### Three-dimensional (3D) co-culture assay

The directional migration of SACC-83 cells and SCs were observed using a modified 3D co-culture models 12, 13. The suspensions of SACC-83 cells (5×10^3^/cm^2^) or SCs (5×10^3^/cm^2^) were mixed with extracellular matrix (ECM gel, Sigma-Aldrich, catalog number: E1270). The ECM connecting bridge was used to generate an attractive chemical gradient. NT-3 (20 ng/ml) was used to mimic the NT-3 expression and AZD7451 (3 nM) 14 was used to block the TrkC.

### CCK8 proliferation assay

SACC-83 cells and SCs were seeded at the density of 2×10^3^ cells per well onto the 96-well plates and then incubated with the conditioned medium for 48 h. For the preparation of the SCs conditioned media, SCs were cultured with serum-free RPMI-1640 supplemented with 0.1% bovine serum albumin (BSA) for 48 h. For the preparation of the SACC-83 conditioned media, SACC-83 cells were incubated with serum-free RPMI-1640 supplemented with 0.1% BSA for 48 h. The cell proliferation was evaluated using the CCK8 kit (CCK, 7sea Pharmatech Co., Ltd) according to the manufacturer's instructions. The OD value was measured at 450 nm.

### Flow cytometry assay

The fluorescein Annexin V-FITC/PI double labeling (BD Biosciences, San Diego, USA) was employed to measure the apoptosis of SACC-83 cells and SCs. Flow cytometry was performed by Cytomics^TM^ FC 500 (Beckman Coulter, USA).

### Patients and specimens

The present research was approved by the Medical Research Ethics Committee of the Fourth Military Medical University, and the informed consent was obtained from all the patients. The present research was approved by the Medical Research Ethics Committee of the Fourth Military Medical University. 78 primary SACC cases and 25 normal salivary gland controls between 2007 and 2011 were obtained from our affiliated hospital tissue bank. All the patients underwent curative surgical resection without prior chemotherapy or radiation treatment, and were diagnosed with SACC based on H&E staining. All SACC patients were followed up by telephoning or medical records. Clinicopathologic parameters including tumor stage, histological type, PNI, and distant metastasis were obtained from medical records and the follow-up results.

### Immunohistochemical staining

Paraffin-embedded tissues were collected for immunohistochemical staining. Polyclonal rabbit anti-human NT-3 (GeneTex, GTX38542, 1: 200), TrkC (GeneTex, GTX54858, 1: 200) was used as the primary antibodies and peroxidase-conjugated goat anti-rabbit IgG antibody used as the secondary antibody. The primary antibodies were omitted in the negative control. All the stained sections were blindly assessed by two independent, experienced pathologists. The staining intensity was divided as follows: weak = 1, intensive = 2. The positive staining rate of tumor cells was < 10% = 0, 10-50% = 1, > 50% = 2. The intensity of staining and the percentage of positive cells were assessed in 5 random fields (400× magnification). The final score was represented as the dyeing intensity multiplied by the positive staining rate. Negative (-), score 0; Low expression (+), score 1 or 2; high expression (++), score 4.

### Statistical analysis

All the *in vitro* experiments were performed in triplicate. *Student's t-test* and *one-way ANOVA* were used to compare the results. The expression evaluation in the SACC and normal salivary gland tissues were performed by the *Fisher's exact test*. The correlation among NT-3, TrkC, and clinicopathologic parameters were measured by *Spearman's rank correlation coefficient test*. *Kaplan-Meier method* was performed for survival analysis, and the *Log-rank method* was used for comparison. The SPSS 24.0 software (IBM, Armonk, NY, USA) was used for the statistical analysis, and *P* < 0.05 was considered statistically significant.

## Results

### Expression of NT-3 and TrkC in the interaction between SACC cells and SCs

**Figure 1:**
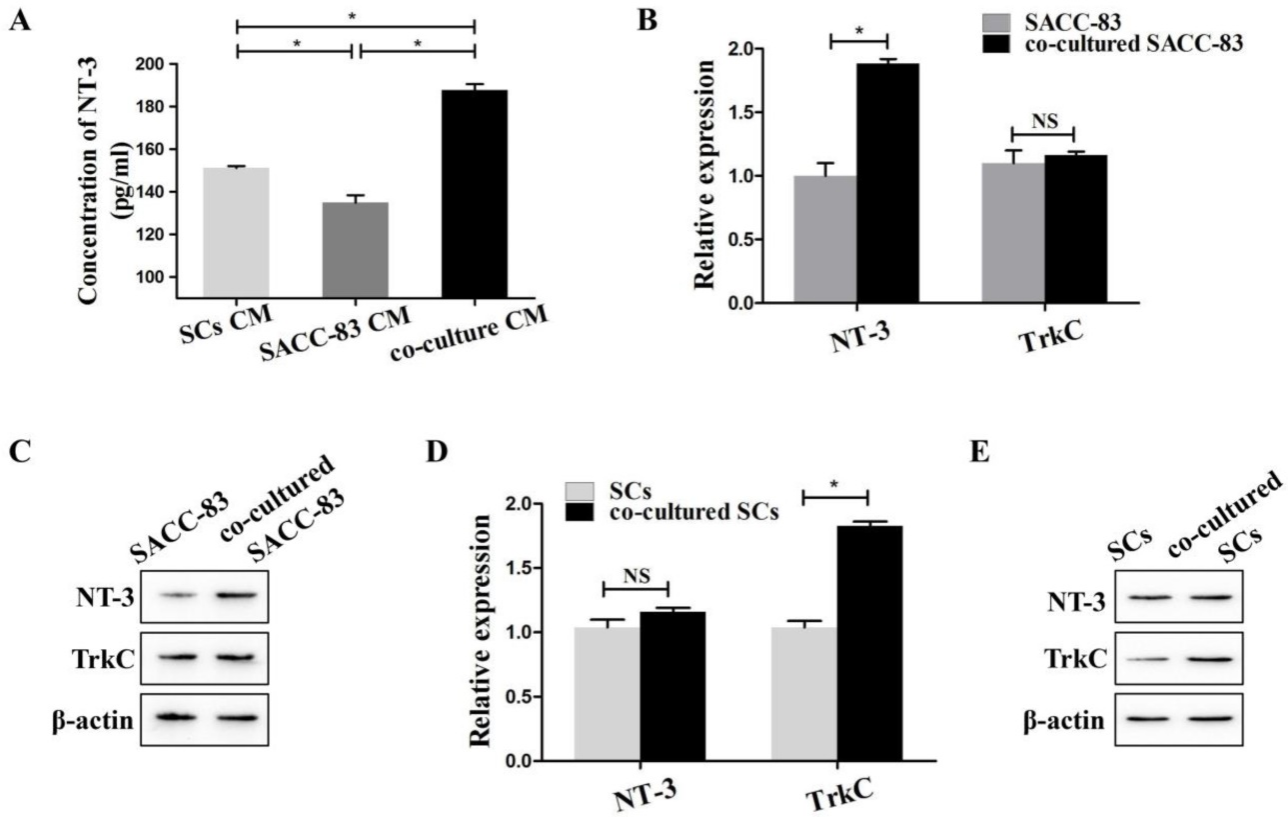
** Expression of NT-3 and TrkC in the interaction between SACC-83 cells and SCs.** (A) The concentrations of NT-3 in the medium of co-cultured cells, solely cultured SACC-83 cells or SCs were detected by ELISA. NT-3 in the co-cultured conditional medium was significantly increased when compared with the medium of the solely cultured SACC-83 cells or SCs. The results of qRT-PCR (B) and western blot (C) showed that the expression of NT-3 was significantly up-regulated in the co-cultured SACC-83 cells than that in the solely cultured SACC-83 cells. The gene (D) and protein (E) expression of TrkC in the co-cultured SCs was significantly higher than that in the solely cultured SCs. *P < 0.05; NS, not significant.

To simulate the interactions between SACC-83 cells and SCs, SACC-83 cells were co-cultured with SCs using the transwell co-culture system. ELISA results showed that the concentration of NT-3 in the co-cultured conditional medium was significantly higher than that in the conditional medium of solely cultured SACC-83 cells or SCs (*P* < 0.05) (Figure 1A).

The results of qRT-PCR (Figure 1B) and western blot (Figure 1C) showed that the expression of NT-3 was obviously up-regulated in the co-cultured SACC-83 cells than that in the solely cultured SACC-83 cells (*P* < 0.05). The expression of TrkC in the SACC-83 cells exhibited no significant changes when cultured alone or co-cultured with the SCs (*P* > 0.05). The expression of NT-3 in the SCs exhibited no significant changes when cultured alone or co-cultured with SACC-83 cells (*P* > 0.05). However, the expression of TrkC was markedly elevated in the co-cultured SCs than that in the solely cultured SCs (*P* < 0.05) (Figure 1D and E).

### Cells motor ability characteristics during the interaction between SACC cells and SCs

**Figure 2:**
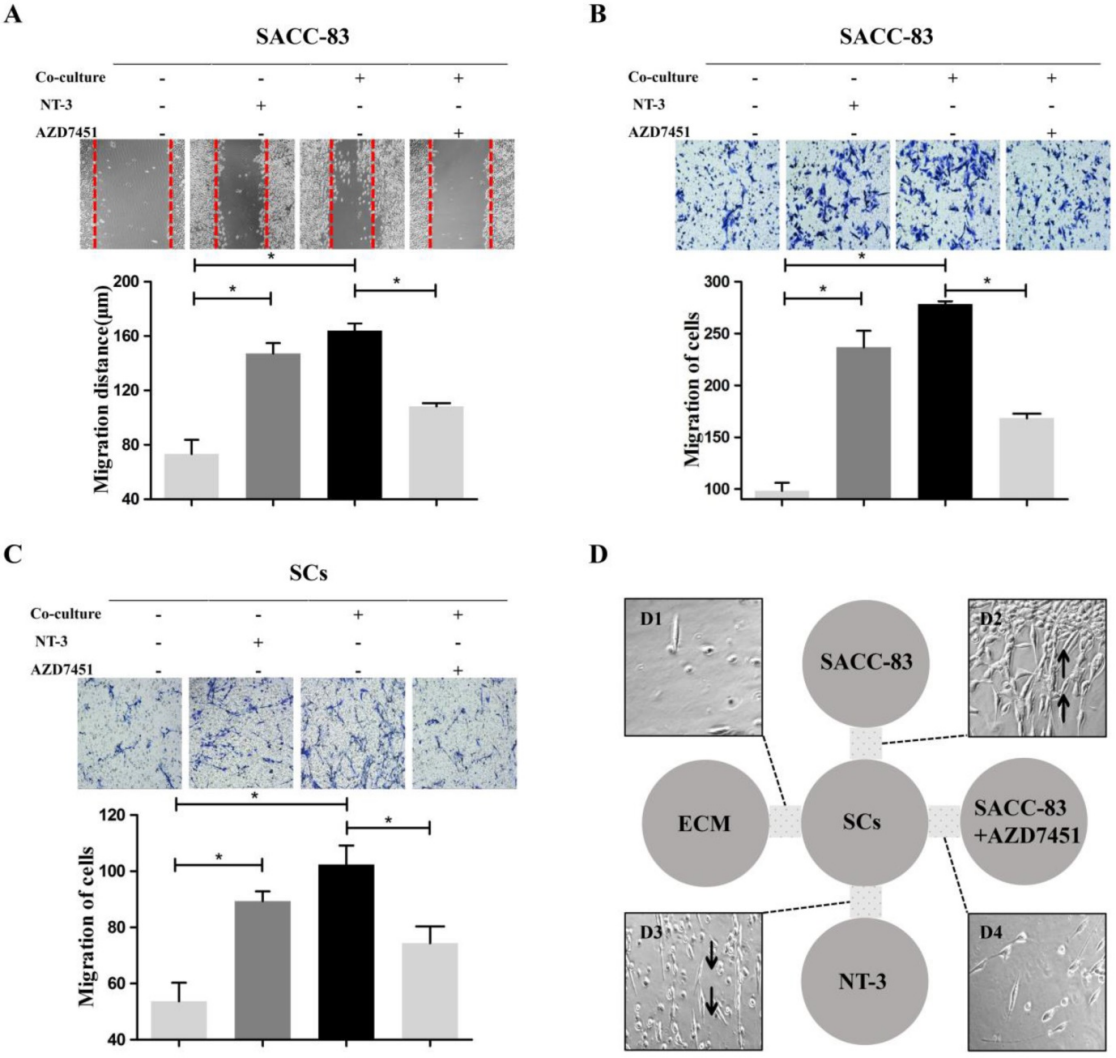
** Effects of the NT-3/TrkC axis on cells motor ability characteristics in the interaction between SACC cells and SCs.** Scratch wound healing assay (A) and migration assay (B, C) demonstrated that the migration ability of the co-cultured cells and the NT-3 treatment group were significantly higher than that of the solely cultured group. On the contrary, AZD7451 inhibited the motility of SACC-83 cells and SCs significantly. *P < 0.05. 3D co-culture assay (D) showed that a stream of cells formed by SCs migrated to the SACC-83 cells (D2). Meanwhile, a stream of cells formed by SCs migrated to the region with a high concentration of NT-3 (D3), with very few SCs migrating to the control group (D1) or the SACC-83 cells with AZD7451 (D4).

Scratch wound healing assay and migration assay indicated that the migration ability of the SACC-83 cells was significantly increased when co-cultured with SCs or treated with NT-3 (20 ng/ml) (*P* < 0.05) (Figure 2A and B). The migration ability of SCs was significantly increased when co-cultured with SACC-83 cells or treated with NT-3 (20 ng/ml) (*P* < 0.05) (Figure 2C). Additionally, inhibition of TrkC by AZD7451 significantly impeded the motility of the SACC-83 cells and SCs even under the co-culture condition (*P* < 0.05) (Figure 2A, B and C).

The modified 3D co-culture model was performed to observe the directional motor ability during the interaction between SACC-83 cells and SCs. It was observed that, before the attack of SACC-83 cells on SCs, a stream of cells formed by SCs migrated towards SACC-83 cells region (Figure 2D2) or the high NT-3 region (Figure 2D3). However, no directional migration of SCs was observed in the negative control group (Figure 2D1) or the SACC-83 cells with AZD7451 group (Figure 2D4).

### Cells proliferation and apoptosis in the interaction between SACC cells and SCs

**Figure 3:**
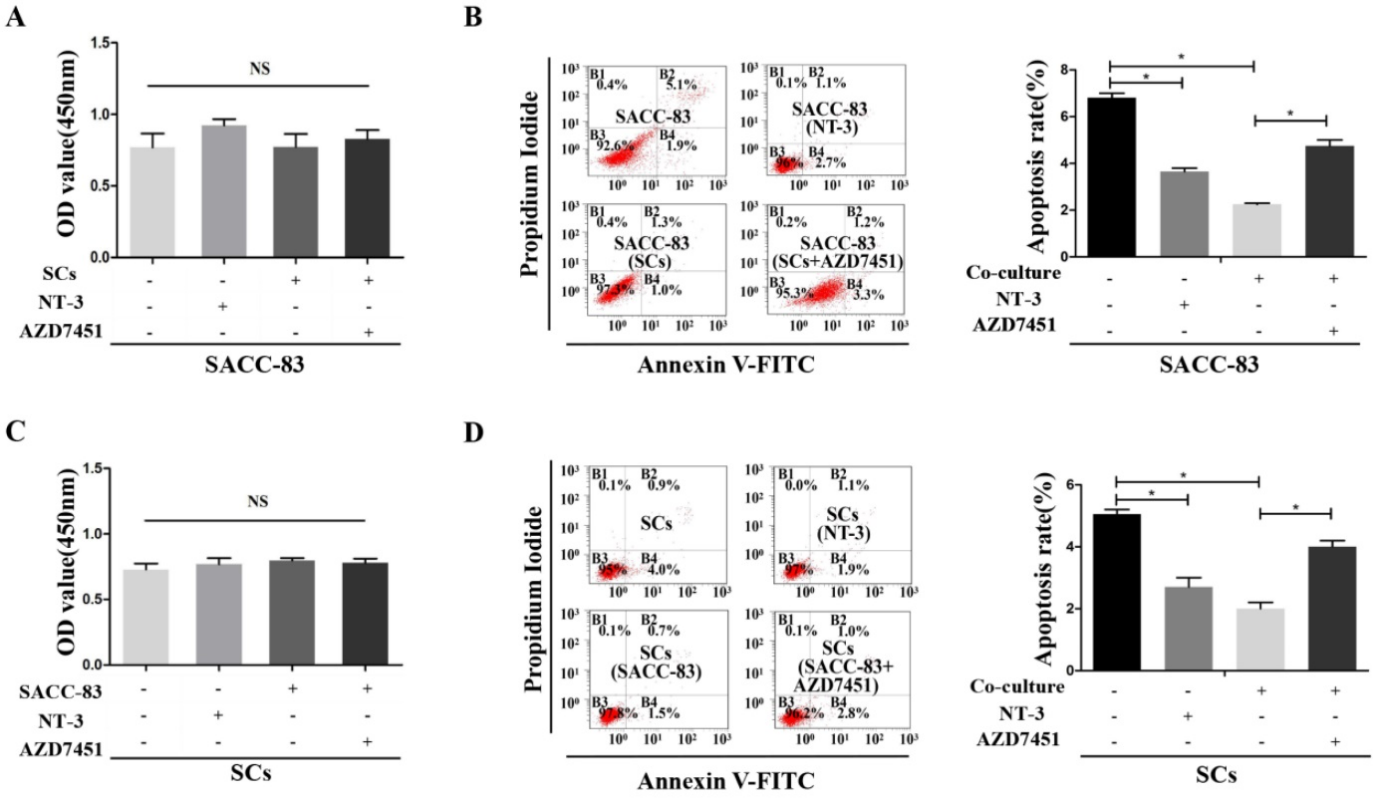
** Effects of the NT-3/TrkC axis on cells proliferation and apoptosis in the interaction between SACC cells and SCs.** The proliferation of SACC-83 cells and SCs were measured by CCK8 assays (A, C). There was no significant difference among the four groups. The apoptosis rate of the SACC-83 cells and SCs were decreased when treated with NT-3 or under the co-cultured condition, while AZD7451 treatment could enhance the apoptosis rate of cells even under the co-cultured condition (B, D). *P < 0.05. The sum of the right upper quadrant (late apoptotic cell) and the right lower quadrant (early apoptotic cell) was calculated as the final apoptosis results.

CCK8 proliferation tests showed that the proliferation of SACC-83 cells was not effected when co-cultured with SCs or treated with NT-3 (*P* > 0.05) (Figure 3A). The apoptosis rate of the SACC-83 cells was decreased when co-cultured with SCs or treated with NT-3, while AZD7451 treatment could enhance the apoptosis rate of SACC-83 cells even under the co-cultured condition (*P* < 0.05) (Figure 3B). In addition, co-cultured condition and NT-3 treatment had no effect on the proliferation of SCs (*P* > 0.05) (Figure 3C). The apoptosis rate of SCs was significantly decreased when co-cultured with SACC-83 cells or treated with NT-3, while AZD7451 treatment could enhance the apoptosis rate of SCs even under the co-cultured condition (*P* < 0.05) (Figure 3D).

### Correlation between the NT-3 and TrkC expression and PNI in SACC

**Figure 4:**
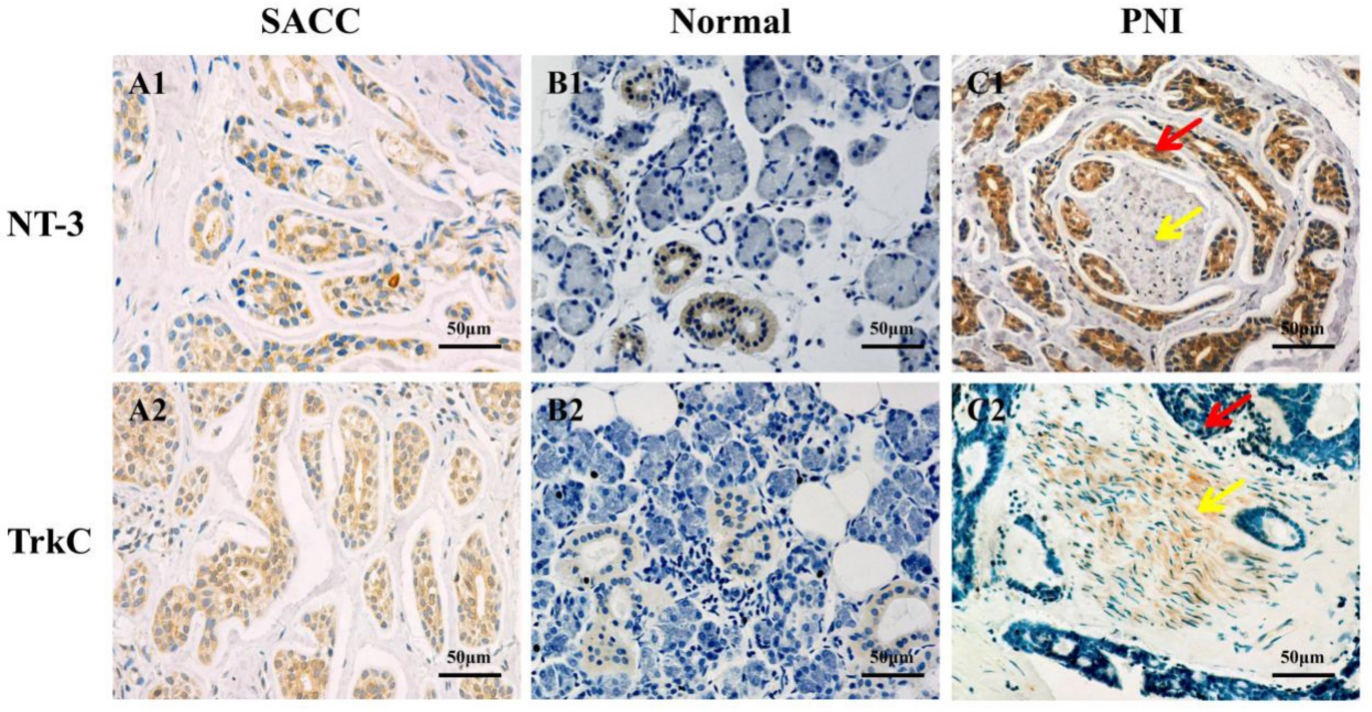
** Difference expression of NT-3 and TrkC in SACC tissues and normal salivary gland tissues.** NT-3 and TrkC were mainly expressed in the cytoplasm of tumor cells (A1, A2). NT-3 and TrkC staining were only detected in some tuber cells of normal salivary glands (B1, B2). NT-3 was highly expressed in tumor cells around the peripheral nerve tissues (C1). TrkC was highly expressed in the nerve invaded by tumor cells (C2). The red arrow represents the tumor, and the yellow arrow represents the nerve.

The expression of NT-3 and TrkC in SACC and normal salivary glands tissues were evaluated by immunohistochemistry. NT-3 and TrkC were mainly expressed in the cytoplasm of tumor cells (Figure 4A1 and A2), but NT-3 and TrkC staining were only detected in some tuber cells of normal salivary glands (Figure 4B1 and B2). Interestingly, the staining intensity of NT-3 in the tumors around the peripheral nerve tissues was significantly enhanced (Figure 4C1). TrkC was highly expressed in the nerve tissues invaded by tumor cells (Figure 4C2). The NT-3 (88.5%, 69/78) and TrkC (92.3%, 72/78) expression were significantly higher in SACC tissues than those of normal salivary glands tissues (12%, 3/25, *P* < 0.01; 20%, 5/25, *P* < 0.01).

As summarized in Table 2, the expression levels of NT-3 and TrkC in the SACC tissues were both significantly associated with the PNI, distant metastasis, and clinical stage (*P* < 0.05), whereas there is no correlation with gender, age, tumor site, and histological type (*P* > 0.05).

### Correlation between the NT-3 and TrkC expression and the prognosis of patients

**Figure 5:**
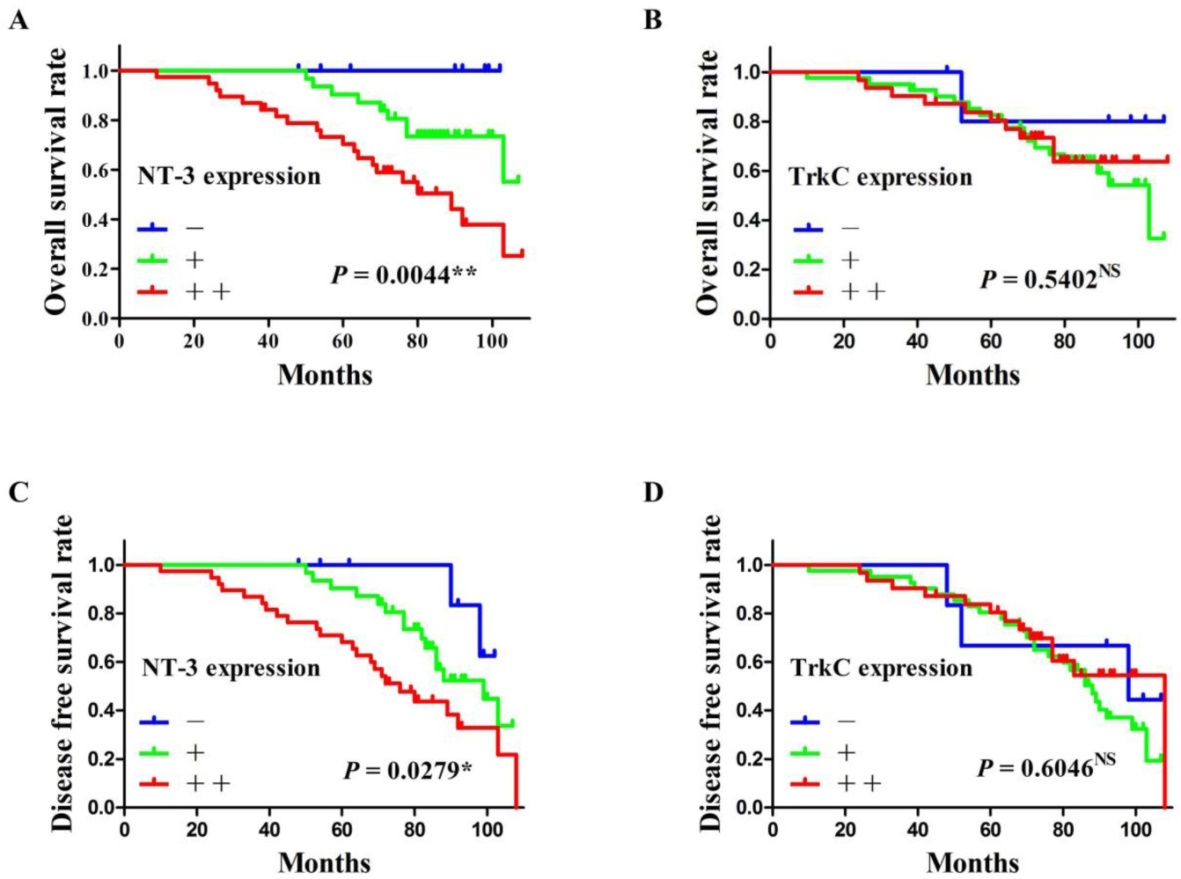
** The survival analysis of SACC patients according to the expression of NT-3 and TrkC.** The high expression of NT-3 was significantly correlated with the overall survival rate and the disease-free survival rate of SACC patients (*P* < 0.05) (A, C). The expression of TrkC was not associated with the overall survival rate or the disease-free survival rate of SACC patients (*P* > 0.05) (B, D).

All patients were followed up until death or more than 5 years. The average follow-up time was 75.06 ± 22.31 months (mean ± SD). At the end of the follow-up, 14 patients (17.9%; 14/78) lost, 43 patients (55.1%; 43/78) alive, and 21 patients (26.9%; 21/78) died. The overall survival rate and the disease-free survival rate of SACC patients were calculated according to the expression of NT-3 and TrkC. As shown in Figure 5, the high expression of NT-3 were significantly correlated with the poor prognosis of SACC patients (*P* < 0.05) (Figure 5A and C), whereas the expression of TrkC was not associated with the prognosis of SACC patients (*P* > 0.05) (Figure 5B and D).

## Discussion

Perineural invasion (PNI) is the notorious biological characteristic of SACC that result in poor prognosis 1, 4. Several PNI related molecules have been proved to be associated with the poor prognosis of SACC patients 15-18. The present study found that the high expression of NT-3 and TrkC were both significantly correlated with the PNI of SACC. And the high expression of NT-3 was significantly correlated with the overall survival rate and the disease-free survival rate of SACC patients. Interestingly, the staining intensity of TrkC was highly expressed in the nerve tissues invaded by tumor cells, while the staining intensity of NT-3 was markedly enhanced in the tumor cells near the peripheral nerve tissues. These results indicated that NT-3/TrkC axis was associated with the PNI and the prognosis of SACC patients.

Substantial evidence demonstrated that the PNI microenvironment of SACC contains a variety of cell components. SCs are a specific type of cells wrapping around the axon of neurons in the peripheral nervous system 19, 20. Resent study found that SCs emerged earlier than the tumor cells in the preinvasive stage during the PNI process of pancreatic and colon cancer 7. In addition, the PNI microenvironment around the tumor and nerve is not only favorable to the tumor cell proliferation and invasion but also conducive to the early migration of SCs 4, 5. Thus, we established transwell co-culture model and 3D co-culture model to investigate the interaction between SACC cells and SCs during the PNI process of SACC. The present study for the first time found that SCs emerged actively and played important roles in the PNI process of SACC.

Recent studies demonstrated that the 3D culture system could not only preserve the material structure of the cell microenvironment in vivo but also could reflect the controlled conditions of cells culture 12. A recent study found that a sudden “U-turn” of SCs toward the pancreatic cancer cells instead of their initial vertical outgrowth direction might occur after crosscutting the sciatic nerve 7. Our modified 3D co-culture model demonstrated that a stream of SCs migrated directionally towards SACC-83 cells region or the high NT-3 region before the attack of SACC-83 cells on SCs. These results showed that NT-3 might be the key factor that mediated SCs directionally migrated towards SACC cells during the PNI process of SACC.

NT-3 21 is a soluble small molecule protein of the neurotrophic factor family that could regulate the proliferation and regeneration of various neural cells. Increasing evidence indicated the NT-3 and its receptor TrkC were overexpressed in multiple cancers, including pancreatic cancer 8, 22, colon cancer 23, and also SACC 11. Our present study found that the expression of NT-3 in SACC-83 cells and the expression of TrkC in SCs were significantly increased in the co-cultured condition. It was found that the NT-3/TrkC axis could greatly enhance the migration ability of SCs 10, 24, 25. Our co-culture assays showed that NT-3 significantly promoted the migration ability of co-cultured SACC-83 cells or co-cultured SCs. Our 3D co-culture results showed that SCs migrated towards SACC-83 cells region or the high NT-3 region. Additionally, the TrkC inhibitor AZD7451 could significantly inhibit these above effects. These results suggested that the NT-3/TrkC axis promoted the directional migration ability of SACC cells and SCs during the interaction between SCs and SACC cells.

Cumulating evidence demonstrated that NT-3 could promote the regeneration of axons 26 and enhance the proliferation of various tumor cells 27, 28. In the present study, NT-3 treatment or co-culture condition obviously inhibited the apoptosis of SACC-83 cells or SCs. In addition, the TrkC inhibitor AZD7451 could significantly inhibit these above results. However, the NT-3 treatment or co-culture condition had no effects on the proliferation of SACC-83 cells or SCs. These results indicated that the NT-3/TrkC axis inhibited the apoptosis of SACC cells and SCs during the interaction between SCs and SACC cells.

In conclusion, the present study indicated that the NT-3/TrkC axis mediated the interaction between SACC cells and SCs in the PNI microenvironment. The NT-3/TrkC axis promoted the directional migration and inhibited the apoptosis of SCs and SACC cells, thus regulated the PNI development and resulted in poor prognosis in SACC. Interruption of the interaction between SCs and SACC cells by blocking the NT-3/TrkC axis might be a novel strategy for the anti-PNI therapy for SACC.

## Figures and Tables

**Table 1:** Primers used for real-time PCR analysis

mRNA	Size (bp)	Species	Cells	Primer sequence
NT-3	495	Homo sapiens	SACC	F: GGGCCCGCCAAGTCAGCATT
				R: TATCCACCGCCAGCCCACG
	204	Rattus	SCs	F: AGCCCCCTCCCTTATATCTAAT
				R: TGATCTCTCCCAACACTGTAAC
TrkC	834	Homo sapiens	SACC	F: CTGCGCCTGGAGCACTGCAT
				R: GAGCAGCTCGGCCTCCCTCT
	110	Rattus	SCs	F: AAGAATCCCCACTTGCGTTATA
				R: AAGTTCTGCTCCAGTCTCAATT
β-actin	205	Homo sapiens	SACC	F: TGACGTGGACATCCGCAAAG
				R: CTGGAAGGTGGACAGCGAGG
	164	Rattus	SCs	F: TTCGCCATGGATGACGATATC
				R: TAGGAGTCCTTCTGACCCATAC

F, forward; R, reverse

**Table 2:** The relationship between the clinic pathological parameters and the expression of NT-3 and TrkC

Variables	n	NT-3 expression			r_s_	*P*-value	TrkC expression			r_s_	*P*-value
		-	+	++			-	+	++		
Gender											
Male	27	3	12	12	0.050	0.667	3	14	10	0.128	0.263
Female	51	6	19	26			3	23	25		
Age											
≤50	41	5	18	18	0.093	0.420	4	20	17	0.087	0.448
>50	37	4	13	20			2	17	18		
Site											
Major	31	3	14	14	0.034	0.767	3	16	12	0.107	0.353
Minor	47	6	17	24			3	21	23		
Histotype											
S	24	2	13	9	0.106	0.358	2	14	8	0.142	0.215
C+T	54	7	18	29			4	23	27		
Stage											
I + II	25	3	16	6	0.290	0.010^a^	3	15	7	0.238	0.036^a^
III + IV	53	6	15	32			3	22	28		
Metastasis											
-	50	7	24	19	0.275	0.015^a^	5	27	18	0.244	0.031^a^
+	28	2	7	19			1	10	17		
PNI											
-	46	7	22	17	0.282	0.012^a^	4	26	16	0.229	0.044^a^
+	32	2	9	21			2	11	19		

^a^*p* < 0.05 by Spearman's rank correlation coefficient test S, solid type; C, cribriform type; T, tubular type

## References

[1] Tian Z, Li L, Wang L, Hu Y, Li J (2010). Salivary gland neoplasms in oral and maxillofacial regions: a 23-year retrospective study of 6982 cases in an eastern Chinese population. International journal of oral and maxillofacial surgery.

[2] Amit M, Binenbaum Y, Trejo-Leider L, Sharma K, Ramer N, Ramer I (2015). International collaborative validation of intraneural invasion as a prognostic marker in adenoid cystic carcinoma of the head and neck. Head & neck.

[3] Bradley PJ (2017). Adenoid cystic carcinoma evaluation and management: progress with optimism!. Current opinion in otolaryngology & head and neck surgery.

[4] Amit M, Na'ara S, Gil Z (2016). Mechanisms of cancer dissemination along nerves. Nature reviews Cancer.

[5] Bunimovich YL, Keskinov AA, Shurin GV, Shurin MR (2017). Schwann cells: a new player in the tumor microenvironment. Cancer immunology, immunotherapy: CII.

[6] Shan C, Wei J, Hou R, Wu B, Yang Z, Wang L (2016). Schwann cells promote EMT and the Schwann-like differentiation of salivary adenoid cystic carcinoma cells via the BDNF/TrkB axis. Oncology reports.

[7] Demir IE, Boldis A, Pfitzinger PL, Teller S, Brunner E, Klose N (2014). Investigation of Schwann cells at neoplastic cell sites before the onset of cancer invasion.

[8] Ohta T, Numata M, Tsukioka Y, Futagami F, Kayahara M, Kitagawa H (1997). Neurotrophin-3 expression in human pancreatic cancers. J Pathol.

[9] Weeraratna AT, Arnold JT, George DJ, DeMarzo A, Isaacs JT (2000). Rational basis for Trk inhibition therapy for prostate cancer. The Prostate.

[10] Yamauchi J, Chan JR, Shooter EM (2003). Neurotrophin 3 activation of TrkC induces Schwann cell migration through the c-Jun N-terminal kinase pathway. Proceedings of the National Academy of Sciences of the United States of America.

[11] Ivanov SV, Panaccione A, Brown B, Guo Y, Moskaluk CA, Wick MJ (2013). TrkC signaling is activated in adenoid cystic carcinoma and requires NT-3 to stimulate invasive behavior. Oncogene.

[12] Huang BW, Gao JQ (2018). Application of 3D cultured multicellular spheroid tumor models in tumor-targeted drug delivery system research. Journal of controlled release: official journal of the Controlled Release Society.

[13] Liu T, Lin B, Qin J (2010). Carcinoma-associated fibroblasts promoted tumor spheroid invasion on a microfluidic 3D co-culture device. Lab on a chip.

[14] Tatematsu T, Sasaki H, Shimizu S, Okuda K, Shitara M, Hikosaka Y (2014). Investigation of neurotrophic tyrosine kinase receptor 1 fusions and neurotrophic tyrosine kinase receptor family expression in non-small-cell lung cancer and sensitivity to AZD7451 in vitro. Molecular and clinical oncology.

[15] Shen Z, Li T, Chen D, Jia S, Yang X, Liang L (2014). The CCL5/CCR5 axis contributes to the perineural invasion of human salivary adenoid cystic carcinoma. Oncology reports.

[16] Amit M, Binenbaum Y, Sharma K, Ramer N, Ramer I, Agbetoba A (2014). Analysis of failure in patients with adenoid cystic carcinoma of the head and neck. An international collaborative study. Head & neck.

[17] Panaccione A, Chang MT, Carbone BE, Guo Y, Moskaluk CA, Virk RK (2016). NOTCH1 and SOX10 are Essential for Proliferation and Radiation Resistance of Cancer Stem-Like Cells in Adenoid Cystic Carcinoma. Clinical cancer research: an official journal of the American Association for Cancer Research.

[18] Morris LGT, Chandramohan R, West L, Zehir A, Chakravarty D, Pfister DG (2017). The Molecular Landscape of Recurrent and Metastatic Head and Neck Cancers: Insights From a Precision Oncology Sequencing Platform. JAMA oncology.

[19] Kidd GJ, Ohno N, Trapp BD (2013). Biology of Schwann cells. Handbook of clinical neurology.

[20] Bhatheja K, Field J (2006). Schwann cells: origins and role in axonal maintenance and regeneration. The international journal of biochemistry & cell biology.

[21] Cattaneo E, McKay R (1990). Proliferation and differentiation of neuronal stem cells regulated by nerve growth factor. Nature.

[22] Miknyoczki SJ, Lang D, Huang L, Klein-Szanto AJ, Dionne CA, Ruggeri BA (1999). Neurotrophins and Trk receptors in human pancreatic ductal adenocarcinoma: expression patterns and effects on in vitro invasive behavior. International journal of cancer.

[23] Luo Y, Kaz AM, Kanngurn S, Welsch P, Morris SM, Wang J (2013). NTRK3 is a potential tumor suppressor gene commonly inactivated by epigenetic mechanisms in colorectal cancer. PLoS genetics.

[24] Genc B, Ozdinler PH, Mendoza AE, Erzurumlu RS (2004). A chemoattractant role for NT-3 in proprioceptive axon guidance. PLoS biology.

[25] Yamauchi J, Chan JR, Shooter EM (2004). Neurotrophins regulate Schwann cell migration by activating divergent signaling pathways dependent on Rho GTPases. Proceedings of the National Academy of Sciences of the United States of America.

[26] Shakhbazau A, Martinez JA, Xu QG, Kawasoe J, van Minnen J, Midha R (2012). Evidence for a systemic regulation of neurotrophin synthesis in response to peripheral nerve injury. Journal of neurochemistry.

[27] Louie E, Chen XF, Coomes A, Ji K, Tsirka S, Chen EI (2013). Neurotrophin-3 modulates breast cancer cells and the microenvironment to promote the growth of breast cancer brain metastasis. Oncogene.

[28] Truzzi F, Marconi A, Lotti R, Dallaglio K, French LE, Hempstead BL (2008). Neurotrophins and their receptors stimulate melanoma cell proliferation and migration. The Journal of investigative dermatology.

